# Expression of *REG Iα* gene in type 2 diabetics in Pakistan

**DOI:** 10.1186/s13098-015-0092-6

**Published:** 2015-11-13

**Authors:** Sadaf Saleem Uppal, Abdul Khaliq Naveed, Saeeda Baig, Bushra Chaudhry

**Affiliations:** Department of Biochemistry and Molecular Biology, Army Medical College, Rawalpindi and National University of Science and Technology, Islamabad, Pakistan; Department of Biochemistry, Islamic International Medical College, Riphah International University, Islamabad, Pakistan; Department of Biochemistry, Ziauddin University, Karachi, Pakistan; Department of Biological and Biomedical Sciences, Aga Khan University, Karachi, Pakistan

**Keywords:** Type 2 diabetes, β-Cell regeneration, β-Cell apoptosis, REG Iα

## Abstract

**Background:**

The escalating rate of diabetes’ has prompted researchers around the world to explore for early markers. A deficit of functional β-cell mass plays a central role in the pathophysiology of type 2 diabetes. The *REG* (Regenerating) gene, encoding a 166 amino acid REG protein was discovered in rats and humans which is released in response to β-cells damage and play a role in their regeneration. The objective of this study was to characterize serum levels of REG Iα proteins in type 2 diabetic patients as indicator of β-cell apoptosis as well as regeneration.

**Methods:**

Unrelated type 2 diabetic patients (n = 55) of different age groups and disease duration were recruited from the Medical OPD of PNS Shifa Hospital. Age and sex matched non diabetic controls (n = 20) without family history of diabetes were selected from the same setting. Demographical details were recorded on a structured questionnaire. Biochemical parameters like FBG, HbA1c, TC and TG levels were measured. Serum levels of REG Iα protein were determined by ELISA.

**Results:**

Levels of REG Iα protein were found significantly raised in type 2 diabetic patients compared to controls (p < 001). Patients with short duration of the disease had higher levels of REG Iα as compared to patients with longer duration of the disease. Although the patients were on anti hyperglycemic agents, a positive correlation was found between REG Iα serum levels, FBG and HbA1c levels. Patients with higher BMI had higher levels of serum REG Iα levels. Serum TC, TG and Hb levels showed no correlation.

**Conclusion:**

REG Iα may be used as a marker/predictor of type 2 diabetes especially in the early stages of the disease.

## Background

Pancreatic islet β-cells produce insulin which regulate body glucose metabolism, but their capacity for regeneration is limited, which plays a major role in the pathogenesis of autoimmune type 1 and insulin resistant type 2 diabetes [1]. An insufficiency in functional β-cell mass and their aptitude to secrete insulin is a characteristic feature of type 2 diabetes. Factors, other than renewal crisis, implicated in β-cell death in type 2 diabetes include glucolipotoxicity, amyloid deposits in pancreatic islet and high metabolic demand causing oxidative stress, endoplasmic reticulum stress as well as inflammatory signals such as cytokines production in the islet insulin producing cells have been associated with β-cell death in type 2 diabetes [2]. Future plan of action regarding prevention as well as treatment of diabetes should include such strategies that lead to amplify functional β-cell mass in pancreas [3, 4]. A constant process of demise and restoration of β-cell mass is an ongoing process under physiological conditions [5]. For normal functioning of islets β-cells a crucial balance between these two processes appears to be obligatory. To date no methods for assessment of β-cell apoptosis in disease conditions in humans is available except for post mortem studies. These studies reported a deficit of up to sixty-five percent in pancreatic insulin producing cells in type 2 diabetes [6].

The novel *REG* (Regenerating) gene, encoding an approximately 17 kDa protein was discovered during the screening of cDNA library derived from regenerating and hyperplastic islet in rats and humans [7]. The Reg1 protein, normally a product of acinar cells of exocrine pancreas has mitogenic effect on β-cells of endocrine pancreas and has been shown to improve experimental diabetes in rats [8–10]. Reg1α protein causes replication of DNA in pancreatic β-cells and a receptor for Reg protein was identified that conveys a growth signal of Reg protein for regeneration of β-cells [11, 12]. In *Reg* knockout mice the size of islets were much smaller and they showed a lower proliferative capacity, while a delay in the onset of diabetes was observed in NOD mice carrying the Ins-Reg transgene [13], indicating a possible role of Reg family proteins in β-cell growth and regeneration. Reduction of Reg1 was linked with the pathogenesis of impaired glucose tolerance of diabetes [14], while treatment with Reg1 protein improved the islet β-cells ability to secrete insulin in rat models of diabetes, indicating its role in the pathogenesis of type 2 diabetes.

REG Iα is one of the five members of the human REG family proteins and is encoded by a gene positioned on chromosome 2p12 [4]. REG protein levels are significantly increased in the islets from a diabetic patient, and antibodies against Reg protein which impede proliferation of β-cell are identified in the mouse models of diabetes [15] and in some diabetic patients [16], suggesting its role in the pathogenesis of human diabetes. This evidence led to the present hypothesis that REG proteins expression is enhanced during type 2 diabetes in their effort to regenerate islet β-cells destroyed due to glucolipotoxicity and increased metabolic demand. Therefore, the objective of this project was to characterize serum levels of REG Iα proteins in patients of type 2 diabetes as markers of β-cell apoptosis and regeneration especially in the early stages of the disease.

## Methods

### Subjects

In this case control study unrelated patients (55) with diabetes were selected from the Medical OPD of PNS Shifa Hospital along with non-diabetic (20) controls. Prior to this the Ethical Committee of Army Medical College approved the study protocol. Blood samples (10 ml) were collected after an informed consent for serum analysis from each subject. Blood sample was collected in clot activator tubes and was allowed to clot for 30 min before centrifugation for 30 min at 2500×*g*, 2–8 °C. Serum was removed and stored in aliquots at −20 °C for ELISA and further analysis.

Serum REG Iα protein levels were measured in 55 patients with type 2 diabetes with different age groups and disease duration and 20 normal sex/age matched controls. ADA criteria was used to assess the patients with type 2 diabetes [1]. Blood sample of each study subject was collected in the morning after an overnight fast. Height, weight, and blood pressure were measured. Biochemical analysis of blood was done for FBG, HbA1c, TC and TG.

### Quantification of serum REG Iα

The REG Iα protein was measured in serum of type 2 diabetics using a human REG Iα BioAssay ELISA Kit (*USBiological*, life Science), which is a sandwich enzyme immunoassay for in vitro quantitative measurement of REG Iα in human serum, plasma and other biological fluids. Samples were diluted 1:500 with 0.01 M PBS as follows: 20 μl of sample were added to 180 μl of PBS (dilution 1:10); 10 μl of this dilution were pipetted into another 490 μl of PBS to prepare the 1:500 dilutions as per requirement. In the BioAssay Human REG Iα ELISA, 100 μl of blank (PBS), standards and samples were added (in duplicates) in the wells of the microtiter strips coated with REG Iα specific antibody. The microtiter plate was incubated at 25 °C for 2 h. 100 µl of the Detection Reagent A working solution (Biotin-conjugated antibody specific to REG Iα) was added to each well and incubated for 1 h at 37 °C. Wells were washed three times with 1× wash solution. 100 μl of the Detection Reagent B working solution [Avidin- conjugated with horseradish peroxidase (HRP)] was added to the wells and incubated for 30 min at 37 °C. Wells were washed five times with wash solution and incubated with 90 μl TMB substrate for 15 min at 37 °C. After adding Stop Solution, reading was done at wavelength of 450 nm with a Microplate reader/spectrophotometer. A standard curve was created by plotting mean absorbance for each standard verses REG Iα concentrations of the standards. The results are presented as REG Iα concentration (pg/ml) in the samples. The amount of REG Iα in the blood sample of the patients was calculated by multiplying the result obtained by dilution factor 500 (for example, 62.5 pg/ml × 500 = 31,250 pg/ml). Readings were converted to ng/ml after multiplying by 0.001 (31,250 × 0.001 = 31.25). The antibodies used in this assay are specific for human REG Iα with no known cross reactivities to recombinant human REG proteins.

### Statistical analysis

Descriptive and inferential statistics were calculated. To check the normality of the data Shapiro–Wilk test were applied. Mean ± SD OR median (inter quartile range) were calculated. The Mann–Whitney U test (for comparison of two groups) and Kruskal–Wallis test (for comparison of 3 or more than three groups) was used to compare quantitative variables. Spearman’s rank test was used for analysis of correlation between variables. Unless otherwise indicated, p values of less than 0.05 were considered significant. Statistical analysis was performed using SPSS statistical software, version 16.

## Results

The clinical characteristics of the study subjects are shown in Table 1. Patients with type 2 diabetes had higher mean BMI, higher systolic blood pressure, higher FBG, HbA1c, fasting TC and TG levels as compared to the control subjects.

**Table 1:** Clinical characteristics of the study subjects

Parameters	Control (n = 20)	Type 2 (n = 55)	*p* value
Sex, M/F	12/8	40/15	–
Age (years)	51 ± 3	55 ± 9	–
Age at diagnosis of disease (years)	–	48.6 ± 8.5	–
Duration of diabetes (years)	–	7.0 ± 5.8	–
BMI (kg/m^2^)	23.98 ± 4.01	25.5 ± 3.9	<0.032*
Systolic blood pressure (mmHg)	120 (110–120)	130 (120–140)	<0.001**
Diastolic blood pressure (mmHg)	80 (70–80)	80 (80–90)	<0.001**
FBG (mmol/L)	3.7 (3.5–4.4)	7.8 (6.1–9.5)	<0.001**
HbA1c (%)	5.5 (5.4–5.6)	7.8 (6.9–9.0)	<0.001**
TC (mmol/L)	3.7 (3.2–4.7)	5.1 (4.5–5.6)	<0.001**
TG (mmol/L)	1.15 (0.9–1.3)	1.7 (1.2–2.3)	<0.041*
Hb (mg/dL)	13.2 (12.6–14.2)	12.0 (11.6–12.8)	<0.054

Data are given as mean ± standard deviation for normally distributed variables, and otherwise as medians (inter quartile range). *P* values of BMI was adjusted for age and sex. *P* values of blood pressure, FBG, HbA1c, and lipid profiles were adjusted for age, sex, and BMI

*BMI* body mass index

*P* values for differences between control group and type 2 diabetes patients. (* significant, ** highly significant)

### Serum REG Iα protein levels

**Fig. 1:**
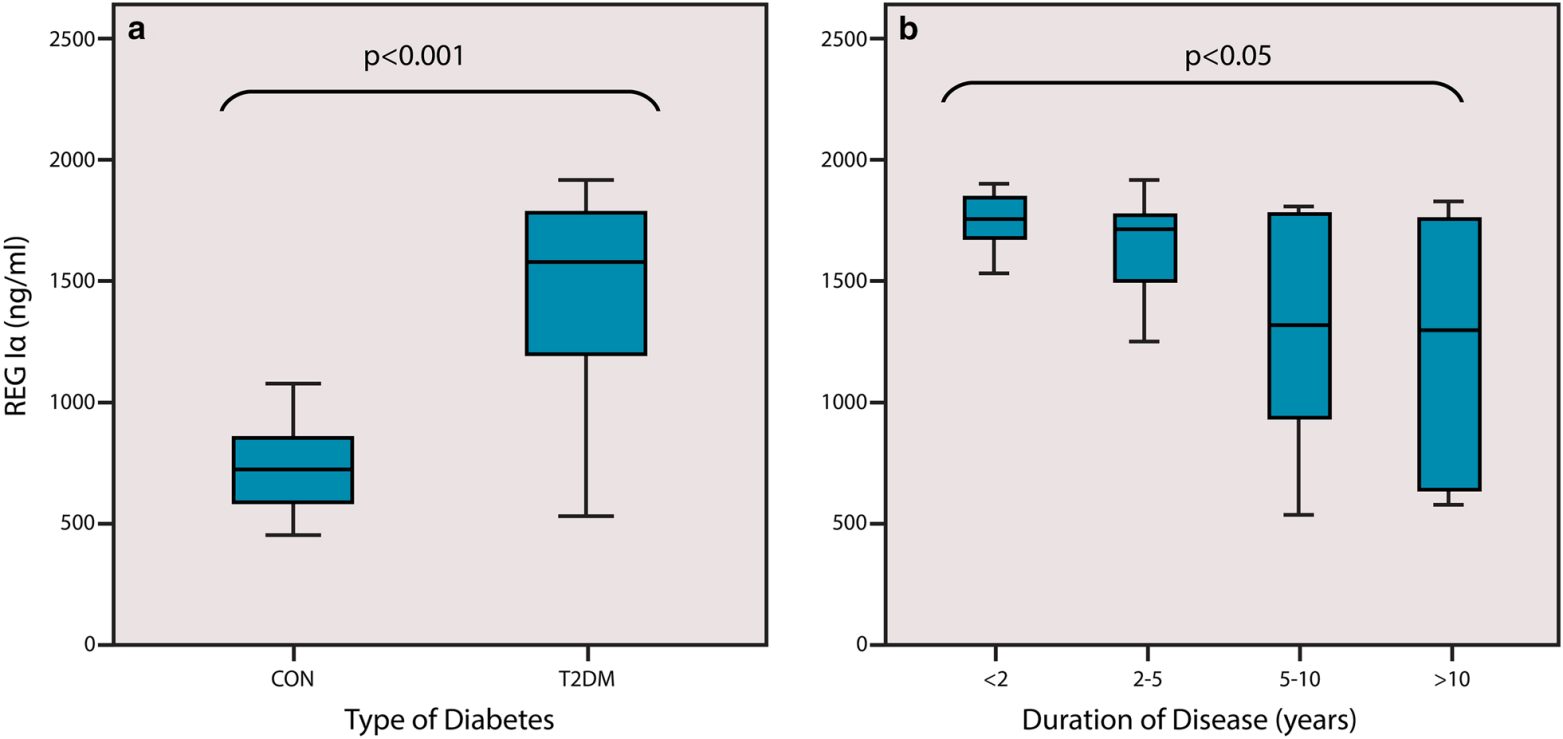
Human REG Iα ELISA assay (*USBiological*, Life Sciences). Quantitative measurement of human REG Iα protein in serum. **a** Increased levels of REG Iα protein were observed in type 2 diabetes patients compared to controls (p < 0.001). **b** Serum REG Iα levels between different groups of type 2 diabetes patients. Significant relationship was found between the groups p < 0.05

Increased REG Iα protein levels were detected in type 2 diabetes patients of different age groups and disease duration compared to age and sex matched controls with a p value of <0.001 (Fig. 1).

### Metabolic and risk factors correlation

**Fig. 2:**
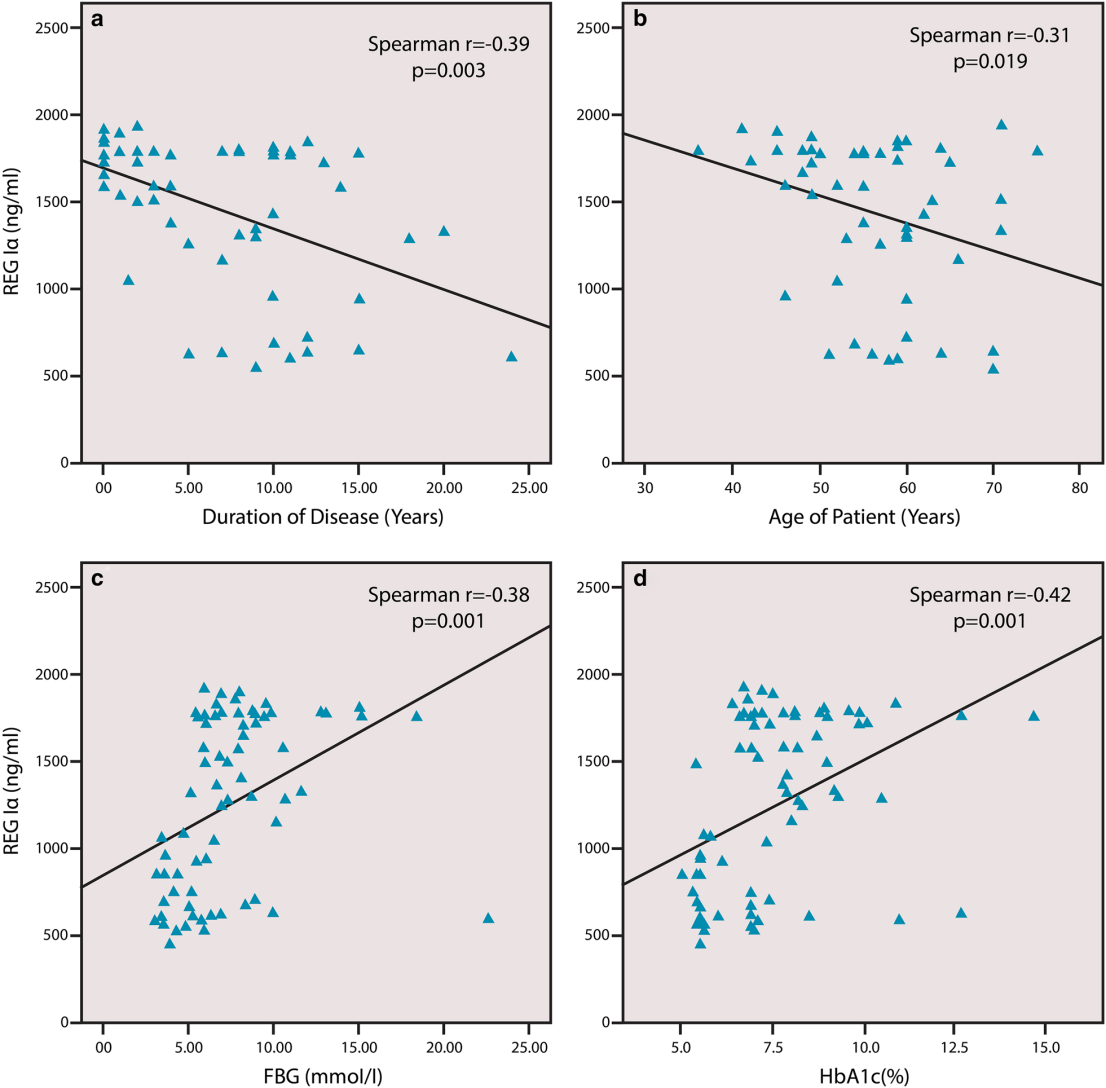
Correlation between clinical characteristics and serum REG Iα protein in type 2 diabetes patients. **a** Correlation between disease duration and serum REG Iα protein. **b** Correlation between age of patient and serum REG Iα protein in type 2 diabetes patients. **c** Correlation between FBG and serum REG Iα protein in type 2 diabetes patients. **d** Correlation between HbA1c and serum REG Iα protein in type 2 diabetes patients

In type 2 diabetics, a negative correlation between disease duration and serum REG Iα protein levels was observed (p = 0.003, Spearman r = −0.39) (Figs. 1b, 2a). Although the protein levels decreased with increase in the duration of the disease they remained significantly higher than the controls. Type 2 diabetes patients were divided into four groups according to the duration of disease. Group 1 (n = 13) patients with duration of disease less than 2 years. Group 2 (n = 14) patients with duration of disease 2–5 years. Group 3 (n = 14) patients with duration of disease 5–10 years. Group 4 (n = 14) patients with duration of disease more than 10 years. Significant difference was found between the four groups with p < 0.05. Between any two groups p value was calculated to be significant or non significant (Group 1 and 2 p = 0.23), (Group 1 and 3 p < 0.05), (Group 1 and 4 p < 0.05), (Group 2 and 3 p = 0.31), (Group 2 and 4 p = 0.15) and (Group 3 and 4 p = 0.63). Serum REG Iα levels also showed a decline in their levels with increasing age of patient (p = 0.019) (Fig. 2b).

Although the patients were on anti hyperglycemic agents, significant positive correlation was found between REG Iα serum levels, FBG and HbA1c levels in type 2 diabetics (Fig. 2c, d). Serum cholesterol, serum triglycerides and hemoglobin levels in type 2 diabetics showed no correlation with circulatory REG Iα levels. Risk factors like smoking and family history of diabetes also did not show any correlation with the REG Iα serum levels in type 2 diabetics. Smokers have slightly higher levels of serum REG Iα compared to the non smokers.

Systolic blood pressure (p = 0.06) and diastolic blood pressure (p = 0.16) were found directly proportional to increase in age, duration of the disease and decrease in REG Iα protein levels.

**Fig. 3:**
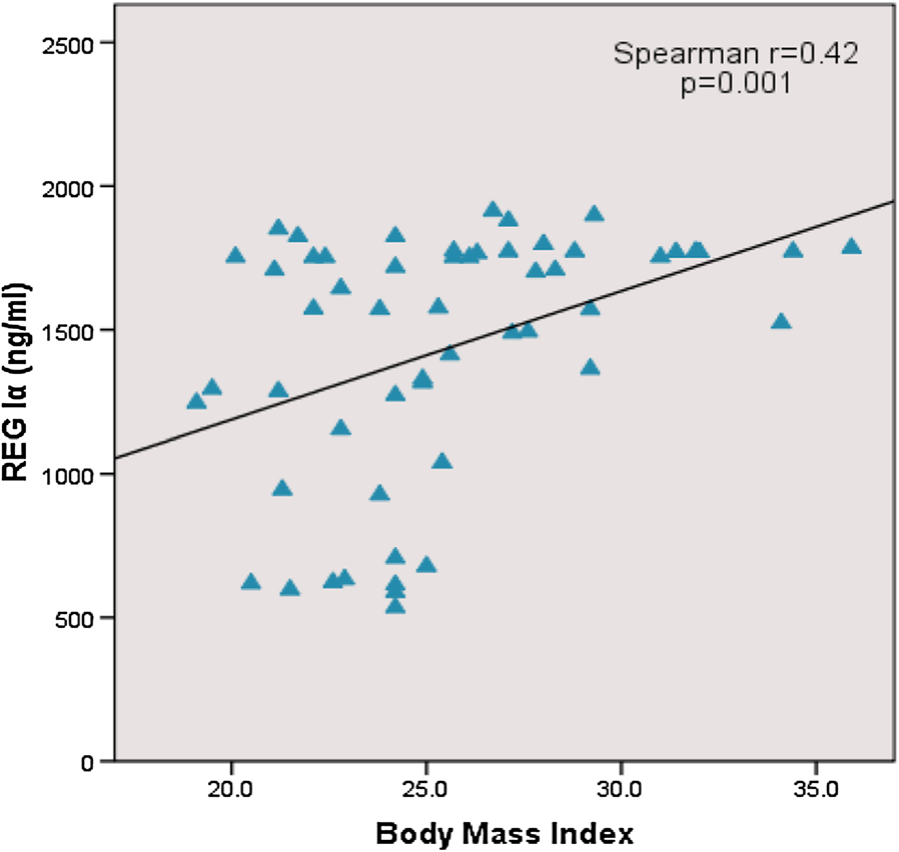
Correlation between serum REG Iα and BMI in type 2 diabetes patients

Patients with increased body weight had higher levels of REG Iα protein with a p value of 0.001 and Spearman r = 0.42 (Fig. 3).

## Discussion

Worldwide there is scarcity of studies regarding role of REG Iα in type 2 diabetes in humans. However, in the developing world where diabetes is becoming a leading cause of death, there are no exploratory studies. Pakistan now ranks seventh in terms of diabetes having 6.9 million cases of diabetes in 2014 according to International Diabetes Federation. A wide range of evidence suggests that a deficit of functional β-cell mass plays a key role in the pathophysiology of diabetes [17, 18] and expression of *Reg* genes following β-cell damage play a role in their regeneration and/or neogenesis [12].

In this study serum levels of REG Iα protein were found significantly increased in type 2 diabetes patient compared to control subjects. The elevated levels of REG Iα in the serum was observed in newly diagnosed patients with type 2 diabetes, and to a lesser degree, in patients with longer duration of the disease. Interestingly, type 2 diabetics with long term disease displayed relatively decreased REG Iα levels and a negative correlation was observed between serum REG Iα levels and disease duration. A negative correlation was also observed between serum REG Iα levels and age of the type 2 diabetes patients.

This study on REG Iα is the first of its kind in type 2 diabetes patients of Pakistani background. Increased circulatory levels of REG Iα in type 1 and type 2 diabetes patient was reported previously in a large scale study conducted in Caucasian population [19]. REG Iα serum levels were also found elevated from third decade of life onwards in patients of maturity onset diabetes of the young (MODY) [20]. Our study also validate the previous widespread work done on experimental models of diabetes and pancreatic β-cell regeneration [13]. *Reg1* expression was also demonstrated to be up regulated at very early stage in high fat diet induced mice models of obesity and type 2 diabetes [21]. This rise is the response of pancreas to hyperglycemia by enhancing the proliferation of pancreatic β-cells and their ability to secrete insulin.

It has been observed that damage to β-cells, due to their infiltration by immune cells, is responsible for up regulation of *Reg1* genes expression [4]. The pathological role of inflammatory mechanism in obesity and type 2 diabetes has been reported by many studies in the last decade [22, 23]. In islets of spontaneous rat models of type 2 diabetes there was increased expression of *Reg1* and *Reg3* in association with peri-islet macrophage infiltration and release of various cytokines/chemokines, particularly IL-6. [24]. Addition of IL-6 and Dx together in human 1.1B4 β-cell lines induced REG Iα and REG Iβ expression [25]. An IL-6 response element has been identified in the promoter region of *Reg1* gene and the local IL-6 levels plays a crucial role in influencing *Reg* gene expression [26]. A recent study has shown that apoptotic β-cells shed microparticles that stimulate expression of *Reg1α* in neighboring cells, to facilitate β-cell regeneration thereby forming a link between β-cell apoptosis and β-cell regeneration. Moreover, the same study also demonstrated that extracellular high glucose concentration potentiates *Reg* gene expression [27]. Increased serum levels of REG Iα protein in patients with type 2 diabetes in present study is in agreement with the hypothesis that the deficit in β-cell mass due to increase in β-cell damage secondary to high metabolic demand and inflammation leads to upregulation of REG Iα in regenerating β-cells and acinar cells.

On the contrary, increased REG Iα levels have also been reported in conditions like Celiac disease [28], ventilator associated pneumonia [29] sepsis [30] and also in other conditions of inflammation and infections, however, the main source of REG Iα remains to be pancreas. Moreover, at the time of sampling all such conditions other than diabetes which lead to raised serum Reg Iα were excluded.

In the present study type 2 diabetics with long term disease demonstrated relatively decreased levels of REG Iα and a negative association was found between serum REG Iα levels and duration of disease. A negative correlation between circulatory REG Iα protein and duration of disease has been previously reported in case of type 1 diabetics [19]. However, no correlation between age or disease onset and REG Iα levels was found in type 1 diabetics while raised REG Iα levels were found in patients with MODY diagnosed in the third decade of life onwards as compared to those diagnosed earlier in life [20]. These patients had much less mean age compared to type 2 diabetics in our study. A recent pilot study conducted by Yang and colleagues reported significant upregulation of *REG I* gene in type 2 diabetes in accordance with our study, however, they related this increase with the clinical stages of the disease and its associated complication [31]. It has been reported that aging is associated with a decline in β-cells proliferation capacity [32]. A decline in the *Reg1* expression has been observed in mice models during the normal aging process and age-related islet dysfunction [33]. In the process of aging and pancreatitis-associated diabetes the decrease in acinar cells, the main source of Reg1 has been demonstrated [14]. In type 2 diabetes with the increase in the duration of disease a reduction in functional β-cell mass occurs [34], thus one-third of patients with long disease duration were found to be on insulin therapy. High intracellular levels of Reg I lead to inhibition of β-cell growth and possible induction of their apoptosis or differentiation into other cell types [35]. Thus, the relative decrease in REG Iα levels with age and duration of the disease may be due to substantial damage to β-cells that occur in late stages of type 2 diabetes, decrease regeneration capacity of β-cells with age and age related decrease in acinar cells, the main source of REG I.

It was however interesting to find that the type 2 diabetes patients with longer duration of the disease and with known diabetic complications were found to have much higher REG Iα levels (data not shown). The exact source of increase serum REG Iα levels is unknown. REG I expression has been addressed as a defense mechanism to the inflammatory response. The other organs damaged/inflamed in these cases may possibly have contributed. Despite the fact that the patients were on anti-hypertensive medication, an increase in the systolic and diastolic blood pressure were observed. In patients with uncontrolled diabetes increased blood pressure is an important risk factor for death and disability [36]. Increased levels of Reg Iα in response to β-cell damage has been associated with β-cell regeneration and increase in their insulin secretory capacity [8], leading to better control of diabetes and its associated risk factors. However, serum cholesterol, triglycerides and hemoglobin levels in type 2 diabetics in this study showed no correlation with circulatory REG Iα levels. Interestingly, a positive though insignificant correlation between REG Iα and smoking was observed. This is possibly due to nicotine in cigarettes, which is associated with apoptosis of β-cells [37] releasing REG Iα.

A significant correlation was found between serum REG Iα, FBG and HbA1c levels in type 2 diabetics; higher REG Iα levels had higher FBG and HbA1c levels. Studies have shown that high glucose concentration potentiates *Reg* gene expression [21, 27]. A recent study conducted by Yang also reported a positive correlation between REG Iα and HbA1c levels in type 2 diabetics [31], while no correlation was found between REG Iα and HbA1c levels in type1 diabetics and in patients with MODY [19, 20]. One of the reasons behind reduced glycemic control in the early stages of the disease was the poor compliance of the patient due to lack of knowledge. Patients with increase BMI had higher serum levels of the Reg Iα protein showing a positive correlation. Relative β-cell mass was demonstrated to be increased in obese versus lean, non diabetic subjects [2, 38]. Obesity associated insulin resistance and hyperglycemia may be responsible for increase in the serum REG Iα levels in this study.

## Conclusion

In this study REG Iα is demonstrated to be significantly upregulated in serum of type 2 diabetes patients, especially in patients of short duration of the disease and may be used as a marker of β-cell damage in type 2 diabetes in the early stages of the disease. Large scale studies in diabetic patients are required with different duration of the disease to further verify the significance of REG Iα protein as an indicator of β-cell regeneration and/or β-cell apoptosis.

## Abbreviations

ADA: American Diabetes Association
Dx: dexamethasone
ELISA: enzyme linked immunosorbent assay
FBG: fasting blood glucose
Hb: hemoglobin
HbA1c: glycosylated hemoglobin
IL-6: interleukin 6
NOD: non obese diabetic
OPD: outdoor patient department
PBS: phosphate buffer saline
TC: total cholesterol
TG: triglyceride
