# Erratum to: Expression of *REG Iα* gene in type 2 diabetics in Pakistan

**DOI:** 10.1186/s13098-016-0124-x

**Published:** 2016-03-07

**Authors:** Sadaf Saleem Uppal, Abdul Khaliq Naveed, Saeeda Baig, Bushra Chaudhry

**Affiliations:** Department of Biochemistry and Molecular Biology, Army Medical College, Rawalpindi and National University of Science and Technology, Islamabad, Pakistan; Department of Biochemistry, Islamic International Medical College, Riphah International University, Islamabad, Pakistan; Department of Biochemistry, Ziauddin University, Karachi, Pakistan; Department of Biological and Biomedical Sciences, Aga Khan University, Karachi, Pakistan

## Erratum to: Diabetol Metab Syndr (2015) 7:96 DOI 10.1186/s13098-015-0092-6

The original version of this article [1] unfortunately contained a mistake in Figure 2. Figure panel 2c and 2d should read as follows:

1. Figure 2c: Spearman r = 0.38 with no −(negative) sign.
2. Figure 2d: Spearman r = 0.42 with no −(negative) sign and p < 0.001 and not p = 0.001.

An updated version of Fig. 2 has been provided below.

**Fig. 2:**
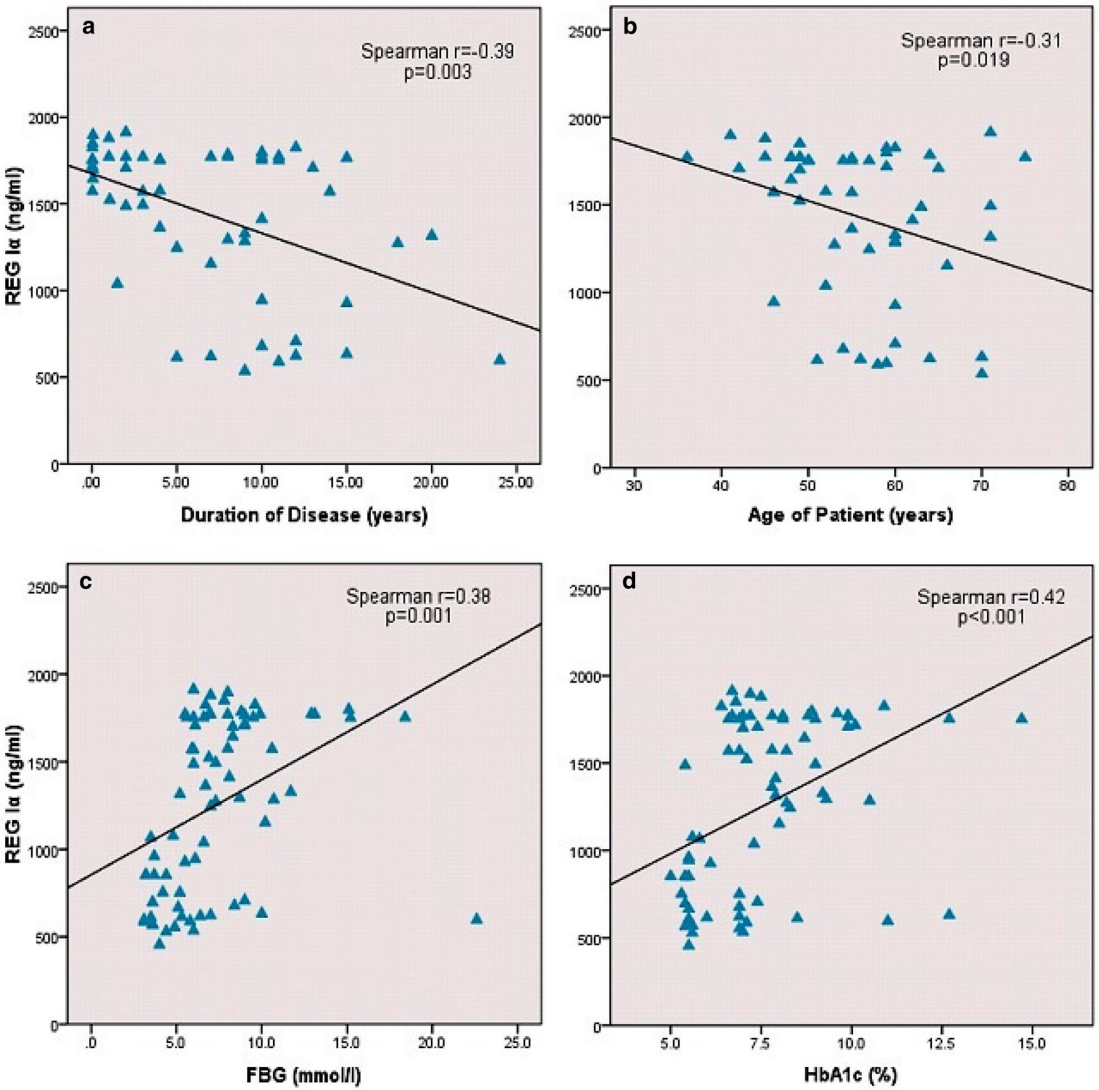
Correlation between clinical characteristics and serum REG Iα protein in type 2 diabetes patients. **a** Correlation between disease duration and serum REG Iα protein. **b** Correlation between age of patient and serum REG Iα protein in type 2 diabetes patients. **c** Correlation between FBG and serum REG Iα protein in type 2 diabetes patients. **d** Correlation between HbA1c and serum REG Iα protein in type 2 diabetes patients

The original version of this article has been updated to reflect this change.
